# Energy Recovery and Economic Evaluation for Industrial Fuel from Plastic Waste

**DOI:** 10.3390/polym15112433

**Published:** 2023-05-24

**Authors:** Ahmed Rida Galaly, Nagia Dawood

**Affiliations:** 1 Department of Engineering Science, Applied College, Umm Al-Qura University, Makkah 24381, Saudi Arabia; 2 Physics Department, Faculty of Science, Taibah University, Al Madina Al Monawara 42363, Saudi Arabia; ndawood@taibahu.edu.sa

**Keywords:** plastic waste, energy recovery, plasma gasification, pyrolysis oil, economical, profits

## Abstract

Plasma gasification is considered an environmentally friendly process to convert plastic waste into fuel oil; a prototype system is described to test and validate the plasma treatment of plastic waste as a strategic vision. The proposed plasma treatment project will deal with a plasma reactor with a waste capacity of 200 t/day. The annual plastic waste production in tons in all regions of Makkah city during 27 years for all months in the years 1994 to 2022 is evaluated. A statistics survey of plastic waste displays the average rate generation ranging from 224 thousand tons in the year 1994 to 400 thousand tons in the year 2022, with an amount of recovered pyrolysis oil; 3.17 × 10^5^ t with the equivalent energy; 12.55 × 10^9^ MJ, and an amount of recovered diesel oil; 2.7 × 10^5^ t with an amount of electricity for sale 2.96 × 10^6^ MW.h. The economic vision will be estimated, using the results of energy generated from diesel oil as an industrial fuel extracted from plastic waste equivalent to 0.2 million barrels of diesel oil, with sales revenue and cash recovery of USD 5 million, considering the sale of each one barrel of diesel extracted from plastic waste in the range of USD 25. It is important to consider that the equivalent barrels of petroleum cost, according to the organization of the petroleum-exporting countries’ basket prices, up to USD 20 million. The sales profit (2022) is as follows: for diesel with a sales revenue of diesel oil, USD 5 million, with a rate of return of 4.1%, and a payback period of 3.75 years. The generated electricity reached USD 32 million for households and USD 50 million for factories.

## 1. Introduction

In Makkah, the holy and largest city in the western region of the Kingdom of Saudi Arabia (K.S.A.), and the most important place on Earth for millions of tourists and Muslim pilgrims from all over the world making the hajj and umrah rituals at least once in their lifetime, due to a large number of pilgrims and human activities, various problems have appeared. Many research groups, during the past forty years, have published with Umm Al-Qura University in the following disciplines, for example: environmental and economic sustainability in the hajj, waste, toxic gases, air pollution, climate change, geophysical and topographic analyses, technological applications, digital transformation, and crowd sciences [1,2,3]. 

**Figure 1:**
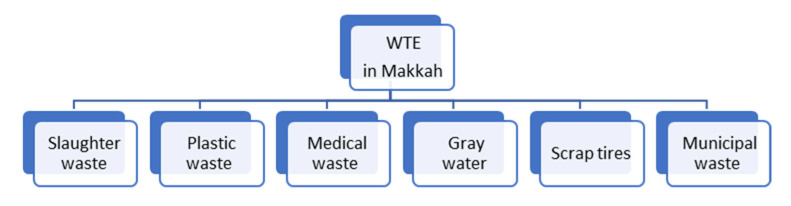
Waste-to-energy visions using the plasma gasification reactor.

Waste is considered a major challenge in the 2030 vision of K.S.A. and plastic waste, especially, is considered the second largest municipal waste stream after scrap tires. The sustainable development of the environment through energy recovery from plastic waste as a source of fuel oil is proposed. This project is one of a series of projects dealing with waste-to-energy (WTE) technology using thermal plasma [4,5], as shown in Figure 1, in Makkah city on energy recovery from municipal waste, scrap tires’ waste, grey water, medical waste, and slaughterhouses’ waste as the perspective work. The current article will cover the energy recovery vision from plastic waste, which represents the second vision of the waste-to-energy series, after our article titled “Treatment of wastes by plasma gasification in Makkah” [6].

There are various ways of managing plastic waste in low-income countries, e.g., landfills, incineration, recycling, and pyrolysis, all of which have many benefits such as financial benefits, job creation, resource conservation, partial economic gains, partial energy saving, and partial environmental protection. On the other hand, there are many disadvantages:

(i) Landfills establish serious risks to the environment and human health, due to the growing amounts of plastic waste, and the high atmospheric temperature in Makkah. As the amount of plastic waste at the lowest depth of the landfill increases, a decrease in the oxygen ratio, degradation, and smoke processes will be produced. In addition, toxic gases such as carbon dioxide, methane, hydrogen sulfide, and dioxins begin to be released, and over time they seep into the ground outside the landfill area [7].
(ii) Recycling has very low economic benefits due to high costs, low profits, unsanitary locations, unsustainable products, and higher energy use, as well as more pollutants [8].
(iii) Incineration, a waste-to-energy process, does not lead to complete combustion; the product is not economical, and therefore has a negative impact as many pollutants, such as nitrogen oxides, sulfur oxides, and fly ash are emitted after the process. In addition, the residual tar and ash represent about 30% of the original volume, and the utilization of energy products resulting from the transformation is inflexible [9].
(iv) Pyrolysis is completely free of oxidants and is a thermochemical conversion process that converts raw materials into char, oil, and combustible gases in an inert atmosphere. A pyrolysis plant does not meet environmental emission standards and causes more air and soil pollution than an incineration plant [10].

In entrained flow gasification with the assistance of streams of hot plasma emerging from the plasma torches [11,12,13], the use of plasma has a considerable influence on the quality of gas, which has a higher heating value of gas. Entrained flow gasification is considered one of the most promising technologies for large-scale, clean, and efficient syngas production at very high temperatures, air oxidation, and various feedstocks. The reaction in entrained flow gasification occurs at a very high rate, with high carbon conversion efficiencies. It has the advantage of adapting to different processes with various feedstocks, such as multi-burner gasification technology with coal, water slurry, non-catalytic partial oxidation technology using natural gas or coke oven gas, and biomass gasification in the early technological developmental phase. Furthermore, water vapor gasification technology can convert solid fuels into cleaner gaseous fuels. Since the water vapor gasification reaction is highly endothermic, high temperatures are normally required to obtain the desired reaction rate. In addition, conventional steam gasifiers require that at least 35% of the feedstock be combusted with air to drive the gasification reaction, resulting in additional NOx emissions.

In recent years, increasingly important energy trends for gasification processes appeared, such as microwave heating technology with high efficiency and easy control. Under the influence of the electric field of an external microwave input, carbonaceous fuels tend to produce nanometer-scale thermal effects in a short time. This method is an increasingly important energy fashion trend [14].

In addition, another new fashion trend for the gasification process is the release of mercury (Hg) into the environment and the distribution of process products. It is of great importance to determine the behavior of Hg in the thermal treatment process of waste, with the parameters’ temperature, residence time, and purge gas flow rate impacting the release of Hg from the waste. Different rates of Hg release during thermal treatment depend on the type of waste. Hg is most readily released from the plastic sample, as there is significant potential to produce low-Hg alternative fuel from waste through thermal treatment [15]. The value of the Hg emission during the combustion process depends on the initial mercury content in the waste, such as coke waste, sewage sludge, coal ash, paper ash, and biomass waste ash with mercury contents of 523.16 µg/kg, 527.81 µg/kg, 6.02 μg/kg, 1.45 μg/kg, and 6.47 μg/kg, respectively. The addition of a polymer up to 10% leads to a reduction in mercury emissions of 53.72% for coal fuels and a reduction of 26.36% in the case of coal sludge [16].

As is known, the four states of matter are solid, liquid, gaseous, and plasma. Plasma can exist in various configurations and can be produced as thermal and non-thermal plasma in technological applications. During the past twenty years, more than 50 articles have been published with Umm Al-Qura University in these disciplines, for example: for cold plasma (non-thermal plasma), the articles [17,18] dealt with sterilization, inactivation of microbes, increasing the performance quality of surgical gown samples, and increasing the antimicrobial performance and the quality of the mechanical properties of Ihram cotton fabric. For hot plasma (thermal plasma) [19,20], our previously proposed phases of the plasma treatment (PTP) projects were as follows: (i) Sustainable development solutions using the plasma treatment project for the medical waste problem to obtain gross profits from the sale of energy products such as syngas, pyrolysis oil, and diesel oil; (ii) Plasma Treatment Project for Municipal Solid Waste to energy (PTPMSW) from the waste source or from a landfill for their sustainable energy, chemical valorization, and the product gas efficiency from waste to gas, and the electrical power generation system was estimated at 5000 kW; (iii) Scrap Tire Plasma Treatment Project (PTPST) to produce synthetic fuels and syngas and hydrocarbons, biofuels, and soot where the main products were 40% slag, 8% synthetic syngas, and pyrolysis oil (52%). After plasma treatment, the annual heat energy produced from scrap tires per ton is 15.5 TJ.

The enormous amount of all kinds and forms of plastic waste requires the rapid development of waste treatment methods on a large scale for an increasingly focused energy recovery (WTE). The use of non-conventional modern methods such as the treatment of plastic waste using plasma reactors differs from the traditional slow methods such as burying, recycling, dumping, or incinerating waste, leading to health and environmental problems and the loss of large amounts of organic matter, which represents 80% of the value of waste [21,22]. The restrictions imposed on traditional techniques and the increasingly strict legislation in the Kingdom regarding waste disposal make plasma technologies the driving force for sustainable development and contribute to the main priority, which is environmental quality. Plasma waste treatment technology is more attractive than other technologies such as recycling, incineration, and gasification because of its environmental benefits and reasonable costs [23,24,25].

The plasma waste treatment technology is content to generate electricity or heat (thermal conversion) and to produce or recover a valuable by-product from the syngas, such as methanol or hydrogen for fuel. The plasma gasification process regarding energy recovery, environmental costs, expected profits, and employment opportunities is analyzed. Either energy efficiency or environmental safety are available in plasma technology as it can handle any chemical composition of waste due to its high temperatures [26,27].

The inorganic components of raw materials are converted into unfiltered lava using a thermal plasma process, while the organic components are gasified and transformed into industrial gases with high calorific values and high material yields. The feedstock is processed to maximize the quality of the synthetic gas using the parameters established by the end user. These conditions (chemical conversion) can be met by syngas which have the highest energy cells. In many facets of everyday life, petroleum is a vital resource for industrial use and massive buildings. When synthetic fuel is generated from plastic treated in a plasma reactor, it costs less than the energy extracted as fuel from petroleum, which is shown as a gross profit [28].

The novelty in the current article, shown clearly in the energy recovery evaluation using plasma reactors, is an economic evaluation model for counting that will be estimated including the cost of the raw material and reactor, revenue expenses, income cash flow, profit, and payback flow for industrial fuel (EEIF) from plastic waste for sustainable development. 

## 2. Materials and Methods

### 2.1. Plastic Waste and Energy Plasma Reactor

**Figure 2:**
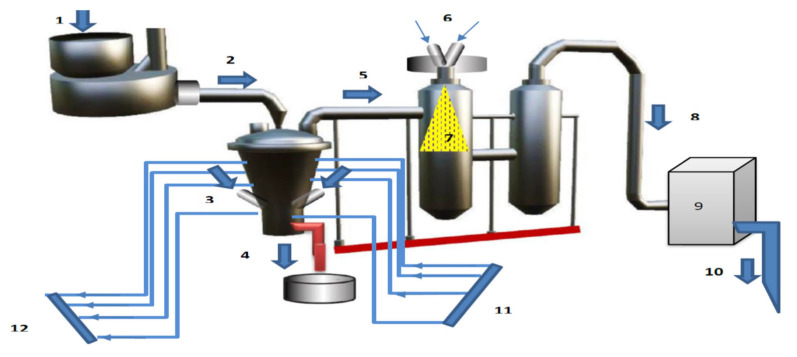
Schematic diagram of the experimental reactor for plasma gasification treatment for plastic waste: 1—plastic chopping; 2—plastic grinding; 3—plasma torch; 4—slag; 5—syngas; 6—air and water in; 7—cylindrical quenching chamber; 8—pyrolysis oil; 9—distillation process; 10—diesel oil; 11—water inlet; 12—water outlet.

The experimental set-up is based on our previously proposed phases of the plasma treatment (PTP) projects for green energy generation in Makkah in the seasons of the pilgrims [29]. Figure 2 shows a plasma treatment reactor design schematic diagram, where the plastic feedstock is dripped into the top of the reactor and forced by gravity through the reactor into the high-temperature plasma zone where it is decomposed and gasified. The reactor chamber is covered with an insulating refractory material that can withstand high temperatures.

The plasma treatment project in the present case study can be represented by four stages as follows: (i) Pre-treatment of plastic waste for rapid processing (such as grinding, chopping, or shredding) before feeding it to the plasma reactor. (ii) Conversion process of plastic waste into three products, i.e., syngas, pyrolysis oil, and slag takes place in a combustion-like chamber at high temperatures of up to 1500–5000 °C. The active working gas is air, which has an important effect on the oxidation in the plasma treatment reactor without the production of environmental pollutants from the produced gasses. (iii) Industrial fuel (pyrolysis oil) is collected, intensified, and purified; the synthetic gas is cleaned; and the end product is the slag, which is periodically or continuously drained at the bottom of the reactor [30,31]. (iv) In the fourth stage, liquid fuels (pyrolysis oil) can be used as an energy product to generate electricity or fuel for consumption in factories such as cement plants [32]. 

The experimental plastic treatment for energy recovery using a plasma gasification reactor is based on the thermal plasma jet coming out of the air plasma torch with an electrical power of 125 kW, an airflow rate of 10–30 mg/s, and temperatures up to between 1500 and 5000 °C. There are two reactor water cooling systems: the first is designed to operate at a wall temperature of up to 1500 °C, and the second is designed to operate with the exiting hot syngas produced and flowing from the furnace reactor through a cylindrical quench chamber. Furthermore, at the exit of the quenching chamber, the gas has a low temperature and enters a condensation chamber.

**Figure 3:**
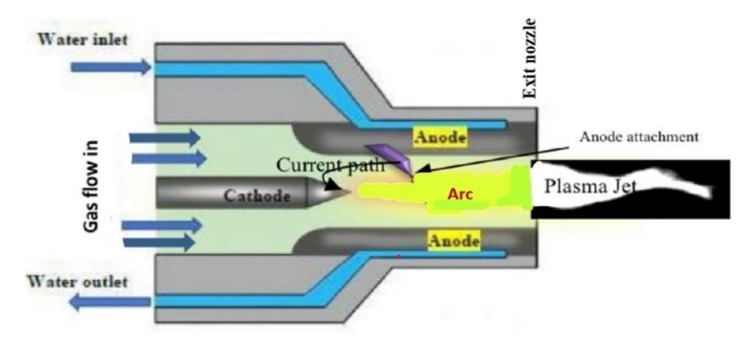
The schematic diagram of the air plasma torch (non-transferred arc).

The air plasma torch is the main component of the plasma reactor, with the parameters given in Table 1. Figure 3 shows the schematic diagram of the air plasma torch (non-transferred arc) powered by a direct current as discussed briefly before in our previous article [33]. The electrical energy is transformed into thermal energy in the plasma torch, where the thermal plasma jet emerges from the torch due to the pressure of the compressed air flow, where there is a fast rate of heat transfer from the plasma torch to plastic waste and the inner walls of the reactor. 

The power, torch type, electrodes, and operating conditions all affect how well plasma chemical reactors operate. Three to five plasma torches will be used for the experiment, allowing us to easily adjust the chemical composition of the plasma and dispose of a variety of by-products [34].

**Figure 4:**
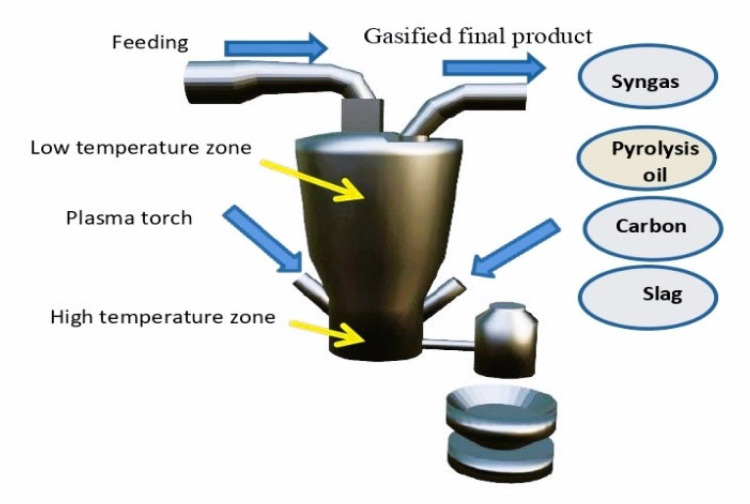
Plasma treatment furnace for WTE process.

Using a plasma treatment furnace with a waste capacity of 200 t/day for the WTE process as shown in Figure 4, with known average plastic waste feeding statistics by tons, and the weight statistics of plastic per year in tons, will help to give information about pyrolysis oil extracted in tons, with equivalent energy by MJ, the amount of extracted diesel oil in tons, and the number of pyrolysis oil gallons. Furthermore, there is a statistical calculation of the amount of electricity sales by MW.h. 

### 2.2. Chemical Conversion and Thermodynamic Process 

The general plasma treatment reaction with air and any waste [35] can be written as (1)

(1) $${\mathrm{CH}}_{x}O_{y}+{\mathrm{wH}}_{2}O+m(O_{2}+3.76\;N_{2})\;\overset{∆}{\to }\;n_{1}H_{2}+n_{2}\mathrm{CO}+n_{3}{\mathrm{CO}}_{2}+n_{4}H_{2}O+n_{5}{\mathrm{CH}}_{4}+n_{6}N_{2}+n_{7}C$$

For our case, where the feedstock plastic waste is represented by CH_x_O_y_ (C_5_H_7.1_O_1.4_), and the moisture content of plastic is represented by H_2_O, the number of moles w is equal to zero, where m and w are the moles of air (O_2_ + 3.76 N_2_) and moisture (water), respectively. The moles of the various plasma treatment products vary from n_1_ to n_7_.

The last equation shows that any organic compound in the waste streams, in the presence of air and temperature, will be converted into the seven products shown. CO and H_2_ are the two main products of interest in the syngas and CH_4_ to a lesser extent [36].

To evaluate the effectiveness and input energy required for the plasma treatment of plastic waste streams, an energy recovery analysis is performed for the parametric investigation of plastic waste streams. The types of plastic with the highest heating value (HHV), or those with the highest levels of carbon and hydrogen, should be delivered to the plasma treatment facility. In our example, the mass product composition (%) could then be calculated for percentage conversion, percentage pyrolysis oil, percentage residue slag, and percentage syngas according to the formula given below from Equations (2)–(5).

(2) $$\mathrm{Conversion}\;\left(\mathrm{wt}\;\%\right)=\frac{\mathrm{mass}\;\mathrm{of}\;\mathrm{the}\;\mathrm{feedstock}\;\mathrm{plastic}-\mathrm{mass}\;\mathrm{of}\;\mathrm{the}\;\mathrm{residue}}{\mathrm{mass}\;\mathrm{of}\;\mathrm{the}\;\mathrm{feedstock}\;\mathrm{plastic}}\times 100\;\%$$

*Liquid Yield:*

(3) $$\mathrm{Pyrolysis}\;\mathrm{oil}\;\left(\mathrm{wt}\;\%\right)=\frac{\mathrm{mass}\;\mathrm{of}\;\mathrm{oil}\;}{\mathrm{mass}\;\mathrm{of}\;\mathrm{the}\;\mathrm{feedstock}\;\mathrm{plastic}}\times 100\;\%$$

*Residue slag (Char) Yield:*

(4) $$\mathrm{Slag}\;\left(\mathrm{wt}\;\%\right)=\frac{\mathrm{mass}\;\mathrm{of}\;\mathrm{slag}}{\mathrm{mass}\;\mathrm{of}\;\mathrm{the}\;\mathrm{feedstock}\;\mathrm{plastic}}\times 100\;\%$$

*Gas Yield:*

Syngas gas (wt%) = 100% − (Oil + slag)**%** (5)

The plasma treatment thermodynamic theory represents the plasma chemical equilibrium processes, and both can be represented by the following efficiency model, which states that *the efficiency* is given as in (6)

(6) $$\eta \;=\frac{\;\mathrm{HEATING}\;\mathrm{VALUE}\;\mathrm{PRODUCED}\;\left(J\right)}{\mathrm{HEATING}\;\mathrm{VALUE}\;\mathrm{FEEDSTOCK}\left(J\right)}\;$$

or as in (7) as follows:

(7) $$\eta =\frac{\mathrm{HHVoil}}{\left(P_{\mathrm{torch}\;}+\;\mathrm{HHVrefuse}\right)}=\frac{\;{ṁ}_{\mathrm{oil}\;}{\;x\;\mathrm{LHV}}_{\mathrm{oil}}}{[{P\;}_{\mathrm{torch}\;}+(\;{ṁ}_{\mathrm{feedstock}\;}{\;x\;\mathrm{LHV}}_{\mathrm{feedstock}})]}$$

where ṁ is the mass flow rate of oil and input plastic waste; P_plasma_ is the electrical power for the plasma torch.

LHV=HHV − 0.212(H + 118 M−0.038 Y) (GJ/T), and HHV = 1.423 [H + 0.214 C − 0.108 Y]

where H is the hydrogen content ratio (%), M is the moisture content ratio (%), C is the carbon content ratio (%), and Y is the oxygen content ratio (%) [37,38].

### 2.3. Economic Evaluation Model for Industrial Fuel (EEIF) Project 

Economic and sale revenues for the conversion process of plastic waste into energy (PWTE) using the plasma gasification reactor represent the third stage of five stages for the sustainable development of plastic waste titled: “Environmental, energy recovery, economic, and profits visions” [39,40]. 

The cost of a plant can be estimated by the cost of raw material (C_RM_), cost of utility (C_UL_), cost of equipment (C_EQ_), cost of labor (C_OL_), cost of waste treatment (C_WT_), investment FCI, and working capital, and then the cost of manufacturing (COM) can be derived as (8):

COM = 0.18 FCI + 2.73 C_OL_ + 1.23 (C_UL_ + C_WT_ + C_RM_) $/year (8)

Considering our products’ targets are diesel oil, pyrolysis oil, and electricity, then the profitability analysis can be derived as follows:

Sale Revenue = The products prices $/year(9)

Considering the following costs:

Cost of Raw Material C_RM_ = $/year (10a)

Cost of Utility C_UL_ = $/year (10b)

Cost of Labor C_OL_ = $/year (10c)

Cost of Waste Treatment C_WT_ = $/year (10d)

Fixed Capital Investment FCI = $/year(10e)

Additionally, the following equation can be used to derive and evaluate expenses, income tax, and the net profit.

The after-tax cash flow (ACF), rate of return (ROR %), total capital investment (TCI), fixed capital investment (FCI), and payback period (PBP) are as follows:

Expenses = (COM + reactors) costs $/year(11)

(12) Income Tax=(Revenue−Expenses) $×(Tax Rate−2.5%)/year

The net profit (PWTE) $ = (Revenue – Expenses − Income Tax)/year(13)

(ACF) $ = (Net profit) $(14)

(15) ROR=(After-Tax Cash Flow/TCI)×100

TCI = FCI + Working Capital(16)

(17) (ACF/TCI)×100=ROR%

where TCI $ > ACF $

TCI $/Net profit $ = (PBP) years(18)

## 3. Results and Discussion 

### 3.1. Vision Regarding Types of Energy Recovery

In Makkah, the treatment of plastic waste using plasma gasification technology is considered a modern method of converting plastic waste into usable energy, providing benefits such as a low proportion of material to be buried, and access to electrical energy through plasma technology.

According to the statistical calculation of the amount of electricity by MW.h., the estimated energy recovery, and the generated electricity, all of these energies can be extracted from plastic waste annually as a renewable and cheaper fuel energy instead of petroleum oil.

A parametric energy recovery analysis of plastic waste streams is performed to assess the output energy represented by (i) pyrolysis oil (tons per year), represented by 0.8 of the input plastic waste, where the typical fuel oil production from pyrolysis (1 kg mixed plastic (type PE, PP, and PS)) is equal to 0.8 kg oil; (ii) the equivalent energy (Megajoule) of the pyrolysis oil equal to 39.6 MJ/kg (Appendix B); (iii) diesel oil (tons per year) represented by 0.85 from the input pyrolysis oil after distillation processes; (iv) the amount of electricity generated (MW.h from the amount of diesel oil), taking into account that 1 MW.h is equal to 3600 MJ. Table 2 shows the output energy of plastic waste from the pyrolysis oil resulting from the plasma gasification process, which will be achieved as the first energy recovery source of a plasma treatment plant. Furthermore, Table 3 shows the output electricity of the diesel oil after the distillation process due to the plasma gasification process, which will be the second energy recovery source [41,42,43], through the Arabic years from 1414 (1994) to 1440 (2022) (Appendix A).

### 3.2. Fuel Type from Plastic Waste Treatment 

After the hot outlet gas is quenched, the pyrolysis oil condensate can be used as a fuel oil, but it cannot be directly used as an energy source due to its high ash and wax content. The collected pyrolysis oil product is very similar to crude oil, brown-black and viscous [44,45,46,47], indicating that it requires many distillation steps to become a source of hydrocarbons in the form of the naphtha product, which improves properties from standard fuel products intended for use in engines. The condensation process of pyrolysis oil at different temperatures produces different types of fuels: (i) gasoline fuel at 500–560 °C, (ii) kerosene fuel at 580 °C and 600 °C, and (iii) diesel oil at 620 °C and 650 °C. 

### 3.3. Energy from Pyrolysis Oil 

**Figure 5:**
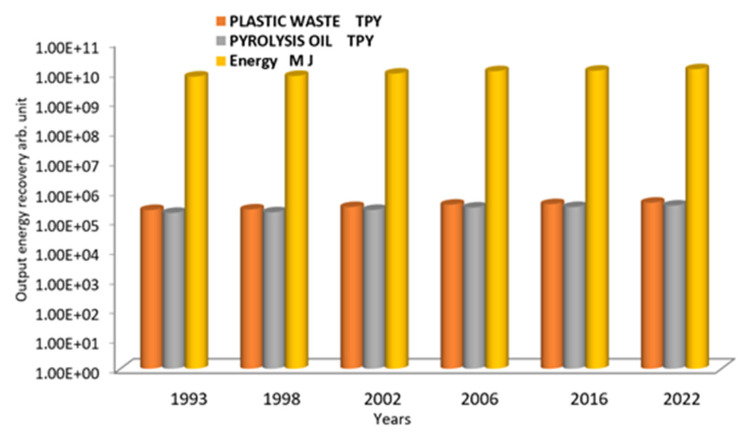
The Logarithmic scale of output energy recovery analysis values of plastic waste using a plasma gasification system.

Using data from Table 2, Figure 5 shows the analysis values of the output energy recovery of waste plastic [47] using a plasma gasification system, showing that fuel oil from the pyrolysis of waste plastic is an important source of the energy product [48,49]. In the pyrolysis process, plastic waste is thermochemically decomposed using a plasma gasification furnace system at temperatures up to 1500 °C and converted into fuel oil, slag, and syngas fractions (oil, solid, and gaseous) [50]. Table 2 shows the total output energy in MJ of the input raw material plastic waste varies from 7.10 × 10^5^ M J in year 1994 to 1.25 × 10^6^ M J in 2022 for input plastic waste ranging from 2.24 × 10^5^ in 1994 to 3.96 × 10^5^ TPY in 2022 and pyrolysis oil ranging from 1.79 × 10^5^ TPY in 1994 to 3.17 × 10^5^ TPY in 2022.

### 3.4. Electricity from Diesel Oil

**Figure 6:**
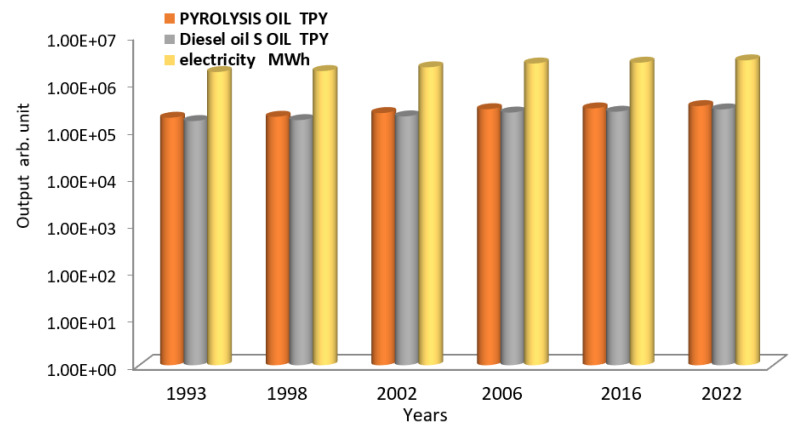
The Logarithmic scale of output electricity produced from the diesel oil after distillation of pyrolysis oil.

Using data from Table 3, Figure 6 shows the output electricity product of diesel oil [51], with lower sulfur and higher cetane numbers compared to traditional diesel [52], after the distillation process for the pyrolysis oil [53,54,55]. Table 3 shows that the amount of electricity generated reaches 1.68 × 10^6^ in 1994 to 2.96 × 10^6^ MW.h in 2022 for pyrolysis oil ranging between 1.79 × 10^5^ TPY in 1994 and 3.17 × 10^5^ TPY in 2022, and the equivalent diesel oil after the distillation process ranging between 1.52 × 10^5^ TPY in 1994 and 2.69 × 10^5^ TPY in 2022.

Even though the destruction of plastic requires more input electricity than other wastes such as paper or wood, plastics undergo the most energy recovery per ton of input feedstock than any other material, mainly because of their greater HHV [56]. 

### 3.5. Economic Evaluation for Industrial Fuel (EEIF)

**Figure 7:**
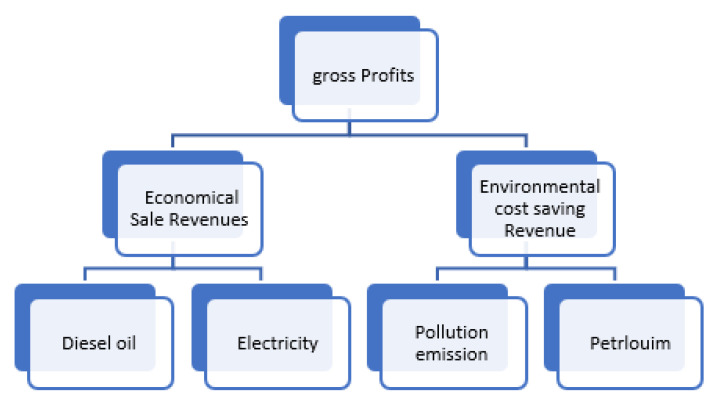
The gross profits of the conversion process of PWTE, represented by economical sale revenues, and environmental cost-saving revenue.

The EEIF from plastic waste is our second fashion target in the current article after energy recovery evaluation, as a sustainable development project achieving vision 2030 in Makkah city. The experimental plasma treatment of plastic waste as an economic vision and the sales revenues of product sales such as pyrolysis oil, diesel oil, and generating electricity, all represent energies that can be extracted from plastic waste annually as a renewable and cheaper fuel energy instead of petroleum oil. By using the project model discussed in Section 2.3, the gross profits of the conversion process of PWTE as shown in Figure 7 will be divided into economical sale revenues, and environmental cost-saving revenue [57] as follows:

#### 3.5.1. Economical Sale Revenues (ESR)

ESR are represented by syngas, diesel oil, generated electricity, coal, and slag. Considering syngas, coal, and slag are the lowest gross profits as shown in our previous articles [Galaly 21, 29, and 33], and our fashion target in the current article is diesel oil and electricity due to their high values, the sales revenues are as follows:

(a) The Sales Revenues of Diesel Oil (SRD)

Table 4 shows the sales revenues analysis of the extracted diesel oil according to the EEIF model annually within 2022, where the average weight of plastic per year is 400 × 10^3^ t, with diesel oil after the distillation process from the pyrolysis oil reaching 0.2 million barrels of diesel oil extracted from plastic waste (BODPW), with the sales revenue of the diesel oil (SRD) as USD 5 million, with ROR as 4.1%, and PBP as 3.75 years, considering the expenses for the five reactors are USD 120 million.

(b) The Sales Revenues of Electricity

Table 5 shows the electricity generation of diesel after the pyrolysis oil distillation process annually within 2022 as follows: (i) diesel reaches 2.7 × 10^5^ t, (ii) the extracted sales amount of electricity reaches 2.96 × 10^6^ MW.h, (iii) the estimated sales profit for generated electricity is up to USD 32 million for households and USD 50 million for factories [58,59] from the five plasma reactors, (iv) the ROR is 26.6% for home (Arafa region), and 41.6% for factories, and (v) the PBP is 2.03 years for home (Arafa), and 1.55 years for factories.

#### 3.5.2. Environmental Cost-Saving Revenue 

The environmental cost-saving revenue (ECSR) can be divided into the following:

(a) Plastic Pollution Emission Cost

Using the air pollution real-time air quality parameters of the Saudi Arabia index and SimaPro program [60,61,62], the results show that the average rate of polluted landfill gas generation reaches 297 thousand megagrams of pollution in 2022 with a cost of USD 7 million annually. This is due to pollution from plastic waste due to the emission of the toxicity of environmental pollutants’ gases arising from plastic waste accumulated in a landfill such as carbon, hydrogen, nitrogen, phosphor, and sulfur which pose a significant threat to human health. Pollution costs, the volume of toxic air pollution, and climate change are decreased to zero emission due to the treatment of plastic waste by the plasma reactor.

(b) Cost-Saving Revenue of Petroleum

The cost-saving revenue of petroleum cashback according to the Organization of the Petroleum Exporting Countries (OPEC) basket price has been explored [63,64,65]. There is a cashback due to the conversion process of PWTE as shown in Table 6. Furthermore, according to the OPEC basket price, the cost-saving revenue of 0.2 million of equivalent barrels of petroleum is USD 20 million (see Appendix B).

### 3.6. K.S.A. Benefits from PWTE Technology

Industrial fuel from plastic waste represents a sustainable development project using plasma reactors according to the Kingdom of Saudi Arabia vision 2030, K.S.A. benefits from PWTE summarized by two benefits: energy recovery, and economic evaluations.

Table 7 shows the comparison between plasma treatment, landfill, and incineration through the process temperature, energy recovery, energy product, public health, climate change, emissions, income, cashback, profits, classification, and K.S.A vision 2030. The thermal plasma processing technique can significantly contribute to the effective conversion of organic molecules into atoms and ions at high temperatures and conversion rates [66,67].

## 4. Conclusions

Based on the obtained results and our previous experiences, it is recommended that the energy recovery for the sustainable development of Makkah city uses WTE technology from plastic waste, which represents our fourth vision related to remapping the waste vision after treatment: solid waste, scrap tires, and medical waste, using thermal plasma reactors as an environmentally friendly solution and renewable energy process to limit global warming without external emissions that are harmful to the environment.

Using the plasma treatment reactors with a waste capacity of 1000 t/day includes the following: the average weight statistics of plastic per year in tons; pyrolysis oil extracted in tons, with equivalent energy by MJ; the amount of extracted diesel oil in tons; the number of pyrolysis oil gallons; and the statistical calculation of the amount of electricity sales by MW.h. Furthermore, the PWTE process satisfies the economic evaluations and environmental sanitation phenomena, through cashback, profits, income for industrial fuel, electricity, and environmental cost-saving revenues. In this paper, the use of thermal plasma is presented as the greenest methodology among plastic management treatments, more than treatments involving the landfill or incineration, thus contextualizing the process in a global waste management system as a non-conventional and renewable energy process.

This project consists of five stages: the current article is the first; the economic vision for PWTE using the plasma reactor is the second; the landfill toxics emission analysis of plastic waste for the environmental vision is the third; the economic evaluation from carbon and slug amounts using PWTE technology and calculating the CO_2_ emitted by the oxidation of the plastic is the fourth; the PWTE technology as a strategic vision is the fifth. The perspective will be to use the same experimental set-up to test and validate the conversion process of slaughterhouses’ waste into usable energy products.

## Figures and Tables

**Table 1:** The plasma torch parameters.

Parameter	Value
Max. voltage	500 V
Max. current	250 A
Max. output power	125 KW
Plasma jet temperature	1500 °C
Max. consumption of air	28 mg/s
Number of torches	Two

**Table 2:** Output energy of plastic waste for Arabic years from 1994 to 2022.

Gregorian Year	Equivalent Arabic Year	Output Energy		
		Plastic Waste TPY	Pyrolysis Oil TPY	Energy MJ
1994	1414	2.24 × 10^5^	1.79 × 10^5^	7.10 × 10^9^
1998	1418	2.35 × 10^5^	1.88 × 10^5^	7.44 × 10^9^
2002	1422	2.82 × 10^5^	2.26 × 10^5^	8.93 × 10^9^
2006	1426	3.40 × 10^5^	2.72 × 10^5^	1.08 × 10^10^
2016	1436	3.54 × 10^5^	2.83 × 10^5^	1.12 × 10^10^
2022	1440	3.96 × 10^5^	3.17 × 10^5^	1.25 × 10^10^

**Table 3:** Output electricity of plastic waste for Arabic years from 1994 to 2022.

Year	Output	
	Diesel Oil TPY	Electricity MW.h
1994	1.52 × 10^5^	1.68 × 10^6^
1998	1.60 × 10^5^	1.76 × 10^6^
2002	1.92 × 10^5^	2.11 × 10^6^
2006	2.31 × 10^5^	2.54 × 10^6^
2016	2.40 × 10^5^	2.65 × 10^6^
2022	2.69 × 10^5^	2.96 × 10^6^

**Table 4:** The sales revenues analysis of the extracted diesel oil according to EEIF model in Makkah annually within 2022.

Parameter	Amount
Capacity of plasma reactor	72 × 10^3^ t
Average weight of plastics	400 × 10^3^ t
Amount of pyrolysis oil extracted	3.17 × 10^5^ t
Amount of diesel oil extracted	2.7 × 10^5^ t
Amount of diesel oil extracted	77.1 × 10^5^ gallons
Energy produced from diesel oil	1.1 × 10^9^ MJ
Amount of diesel oil extracted	0.2 million barrels diesel oil
1BODPW	USD 25
(SRD)	USD 5 million
ROR %	4.1%

**Table 5:** The sales revenues analysis of the generated electricity according to EEIF model in Makkah annually within 2022.

Parameter	Amount
Capacity of plasma reactor	72 × 10^3^ t
Average weight of plastics	400 × 10^3^ t
Amount of pyrolysis oil extracted	3.17 × 10^5^ t
Amount of diesel oil extracted	2.7 × 10^5^ t
Energy produced from diesel oil	10.69 × 10^9^ MJ
Amount of sales of electricity	2.96 × 10^6^ MW.h
Unit cost of electricity sales	USD 12/MW.h for home (Arafa) (2022) USD 17/MW.h for factories (2022)
Sales revenue of electricity	USD 32 million for home (Arafa) (2022) USD 50 million for factories (2022)
ROR %	26.6% for home (Arafa) (2022) 41.6% for factories (2022)
PBP	2.9 years for home (Arafa) (2022) 1.55 years for factories (2022)

**Table 6:** The environmental cost-saving revenue according to EEIF model in Makkah annually within 2022.

Parameter	Amount
Saving 0.2 million BOE cost	USD 20 million
Saving PE cost	USD 7 million
ECSR	USD 27 million
ROR %	22.5%

**Table 7:** Plastic treatment comparison between plasma reactor, landfill, and incineration.

	Items	Plasma Reactor	Landfill	Incineration
**1**	**Process temperature**	Controlled, 1500–5000 °C	The temperature is growing with impossible thermal control	850–1200 °C
**2**	**Energy product**	Industrial fuel, diesel oil, and electricity	Loss of energy due to the emission of toxic gases	Crude oil
**3**	**Energy recovery**	Yes	No	Limited
**4**	**Public health**	Benefit	Harmful	Benefit less than plasma treatment
**5**	**Climate change**	No air pollution	Large volume of toxic	Limited
**6**	**Emissions**	Zero emission	Pollutant emissions	Emissions less than landfill
**7**	**Income and cashback**	Revenue in the short run	Expenses	Revenue in the long run
**8**	**Profits**	High economic evaluation	Negative economic evaluation due to pollution cost	Low economic evaluation
**9**	**Classification**	Non-conventional	Conventional	Conventional
**10**	**K.S.A vision 2030**	Future perspective	Environmental obstacles	Old-fashioned

## Data Availability

Data are contained within the article.

## Appendix A

**Table A1:** Numbers and names of Arabic months and equivalent Gregorian months.

Months Number	Arabic Months	Equivalent Gregorian Months
1	Muharram	January
2	Safar	February
3	RabiulAwwal	March
4	RabiulAkhir	April
5	JamadilAwal	May
6	JamadilAkhir	June
7	Rejab	July
8	Shaa’ban	August
9	Ramadhan	September
10	Shawal	October
11	ZulQaedah	November
12	ZulHijjah	December

## Appendix B

**Table A2:** Values and amount of energy parameters’ units.

Parameter	Amount
1 ton diesel	28.55 gallon
1 MW.h	3600 MJ
1BOE	42 gallon
1BOE	6118.14346 MJ
OPEC basket price	USD 100
